# The Southern Ocean with the largest uptake of anthropogenic nitrogen into the ocean interior

**DOI:** 10.1038/s41598-020-65661-2

**Published:** 2020-06-01

**Authors:** Xianliang L. Pan, Bofeng F. Li, Yutaka W. Watanabe

**Affiliations:** 1 Graduate School of Environmental Science, Hokkaido University, Sapporo, Japan; 2 Faculty of Environmental Earth Science, Hokkaido University, Sapporo, Japan

**Keywords:** Ocean sciences, Marine chemistry

## Abstract

The oceanic external nitrogen (N_ex_) deposition to the global ocean is expected to rise significantly owing to human activities. The Southern Ocean (SO) is an important pathway, which brings external influences into the ocean interior. It touches the borders of several developing countries that emit a large amount of anthropogenic nitrogen. To comprehend the dynamics of N_ex_ in the SO, we developed a new method to assess the change in the oceanic uptake of N_ex_ (ΔN_ex_) in the entire SO. We obtained the spatiotemporal distribution of ΔN_ex_ in the SO by applying this method to a high-resolution grid data constructed using ship-based observations. During the 1990s to the 2010s, N_ex_ increased significantly by 67 ± 1 Tg-N year^−1^ in the SO. By comparing this value with the rate of N_ex_ deposition to the ocean, the SO has received ~70% of N_ex_ deposition to the global ocean, indicating that it is the largest uptake region of anthropogenic nitrogen into the ocean interior.

## Introduction

The reactive nitrogen (N_r_, i.e. NO_x_, NH_y_, and dissolved organic nitrogen) input to the open ocean has increased significantly since 1860, especially in the last two decades^1^. Such consistent increase in the reactive nitrogen input could lead to changes in the ocean nitrogen and carbon cycles apart from affecting the marine biological productivity. Anthropogenic nitrogen released by human activities such as industrial nitrogen fixation and combustion of fossil fuel has contributed the most towards this increase. Nearly 70% of oceanic external nitrogen (N_ex_), which is defined as the input of fluvial and atmospheric N_r_ in this study, is anthropogenic N_r_^2^. Considering that the turnover time of natural N_r_ in the ocean is approximately 3,000 years^3,4^, the change in N_ex_ (ΔN_ex_) on the decadal timescale can closely reflect the change in the anthropogenic uptake in the ocean. The distribution of ΔN_ex_ in the surface ocean has been reported by several studies^1,5,6^. However, the spatiotemporal distribution of ΔN_ex_ in the ocean interior is yet to be revealed clearly; consequently, we lack the comprehension of the amount and storage of anthropogenic nitrogen received by the ocean as well as the variation in the oceanic uptake of anthropogenic nitrogen with time.

The Southern Ocean (SO, south of 30°S) covers approximately 30% of the global ocean surface area, and it is an important pathway that drives external influences such as anthropogenic impact into the global ocean interior owing to the strong movement of water masses (e.g. meridional overturning circulation)^7^. The SO is also very susceptible to anthropogenic materials because much of the sea surface water flowing into the SO touches the borders of several developing countries such as China, India, and South-East Asian countries. Therefore, clarifying the ocean dynamics of N_ex_ in the SO is crucial for gaining a deep understanding of the human impact on the ocean.

However, there are two challenges in exploring the presence of N_ex_ in the SO. One is the difficulty in acquiring ocean observations owing to the severe environmental condition of the SO. Ship-based observational data of the SO are considerably deficient compared with those of other oceans in the Northern Hemisphere. Recent studies on climate change in the SO have mainly focused on multiple repeated ship-based observations along the same lines every decade; the data collected is sparse owing to the difficulty in collecting data from the entire SO^8,9^. The other challenge is difficulty in separating N_ex_ from the internal nitrogen (recycled nitrogen, N_in_). Kim *et al*. (2014) reported the impact of anthropogenic nitrogen on the western North Pacific using N^*^ and the water mass age^6^. Their approach could not remove the effect of nitrogen fixation and denitrification; consequently, it was difficult to estimate the anthropogenic nitrogen in the ocean accurately and apply it to the global ocean.

Recently, a new method capable of estimating the change in anthropogenic CO_2_ impact on the ocean interior across decadal time intervals using parameterization techniques was proposed^10^, which makes it possible to overcome the two abovementioned difficulties related to the SO. Here, we have extended this method to N_ex_ and attempted to obtain the spatiotemporal distribution of ΔN_ex_ in the entire SO.

## Results and Discussion

### Parameterization of reactive nitrogen

We use nitrate (N) to represent N_r_ because nitrate accounts for more than 90% of N_r_ and it is the most stable dissolved form of nitrogen in the interior ocean (where most of ammonium and organic nitrogen are already conversed into N through nitrification or remineralization)^3^. The parameterization technique allows us to reconstruct the nitrate concentration spatiotemporally in the SO by using other hydrographic properties^11^. We used the hydrographic data for dissolved oxygen (DO or O_2_), water temperature (T), salinity (S), and pressure (Pr) along with the observed N (N_obs_) to perform the parameterization of N in the SO. All the data we used were sourced from Global Ocean Data Analysis Project version 2 (GLODAP v2), Climate and Ocean: Variability, Predictability and Change (CLIVAR), and Carbon Hydrographic Data Office (CCHDO) (https://cchdo.ucsd.edu/; Table S1 and Fig. S1(a))^12,13^. By giving several data constraints in obtaining an optimal parameterization (Table S2), we obtained the predicted concentration of N (N_p_) in the SO, as follows:

1 $${{\rm{N}}}_{{\rm{p}}}=394.3\,-\,{9.208\times 10}^{-2}\cdot {\rm{D}}{\rm{O}}-1.534\cdot {\rm{T}}\,-\,9.862\cdot {\rm{S}}+{2.029\times 10}^{-4}\cdot Pr$$

(Number of data points (n) = 65,257; Coefficient of determination (R^2^) = 0.97; Root-mean-square error (RMSE) = 0.80 µmol kg^−1^)

More details of our parameterization method are presented in Figs. S2 and S3 and Table S3. Several statistical tests and an independent dataset were used to confirm the accuracy of our parameterization method (see Supplementary Text S2 for details). Additionally, we compared the spatial distributions of N_obs_ and N_p_ in the SO of 30°S south at surface, 500 m, 1,500 m, 3,000 m and 5,000 m (Fig. S4); consequently, the distribution of N_p_ was in good agreement with that of N_obs_, demonstrating that our parameterization has high accuracy and applicability to the reconstruction of N in the entire SO.

## Oceanic uptake of external nitrogen

### Separation of N_ex_ from oceanic N

N_obs_ comprises an internal term (N_in_) and an external term (N_ex_) because the modern hydrographic data we used were already influenced by changes in the external matter. Heretofore, the separation of these two terms of N_obs_ was challenging. A method to estimate the variation in the external term of the observed ocean carbon species across different arbitrary years was proposed recently^10^. This method could be extended to distinguish N_in_ and N_ex_ (see Supplementary Text S4). We assumed that N_ex_ contained in N_p_ is the average N_ex_ between 2000 and 2016 (N_ex 2008_) and it remains constant with time due to the use of cruise data from 2000 to 2016 for constructing the parameterization of N_p_. We can estimate the variation in N_in_ by considering the difference in N_p_ across different years (ΔN_p_) due to the difference in N_ex_ as zero. The variation in N_ex_ (ΔN_ex_) can be obtained by subtracting ΔN_p_ from the variation in the observed N (ΔN_obs_) (Fig. S6; Eqs. (S2–S5)). Here, N_in_ includes the nitrate originating from the processes associated with DO, T, S, and Pr in the ocean, such as biological nitrogen fixation and remineralization; N_ex_ represents only the effects of atmospheric deposition and riverine nitrogen.

**Figure 1:**
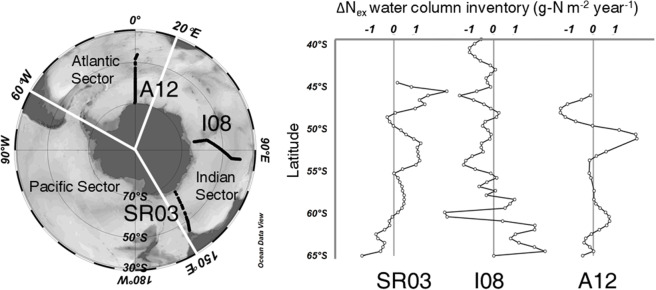
Meridional distributions of water column inventory of ΔN_ex_ along three lines in the SO. SR03 (left, from 1991 to 2011), I08 (middle, from 1994 to 2016), and A12 (right, from 1992 to 2014), as the annual rate of water column inventory of ΔN_ex_ during the period from the 1990s to the 2010s, in units of g-N m^−2^ year^−1^. The inventories were determined by integrating from the surface to the sea floor. White lines separate the three sectors of the SO (the Pacific sector, the Indian sector, and the Atlantic sector). This figure was drawn using Ocean Data View^31^.

Through this method, we noticed that we could estimate ΔN_ex_ for a particular place by using ΔN_p_ along with the data for ΔN_obs_ of that place for different years (Fig. S6(b)). In order to draw the cross sections of ΔN_ex_ in the SO, we selected three repeated observations from the 1990s to the 2010s along the lines SR03, I08, and A12 as the representative data for the Pacific, Indian, and Atlantic basins (Fig. S7). Considering the uncertainty of the N_p_ parameterization (RMSE = 0.80 µmol kg^−1^) and the propagation of uncertainty from the calculation (Eq. (S5)), ΔN_ex_ has an uncertainty of 1.13 µmol kg^−1^, which means that ΔN_ex_ larger than this value must be significant. We estimated the meridional distributions of total water column inventory of ΔN_ex_ along each section (Fig. 1) by integrating ΔN_ex_ from the surface to the sea floor. Both SR03 and A12 have high water column inventories of ΔN_ex_ between the Antarctic Polar Front and the Subantarctic Front (50°S to 55°S), and both I08 and A12 near the Antarctic continent (60°S) also show high water column inventories of ΔN_ex_. Considering the low primary production on the surface of the SO^14^, the N_ex_ deposited on the surface must mainly enter the ocean interior through the formation of intermediate and deep waters and the penetration of surface water mass in the SO^15^. The Antarctic Circumpolar Current has become more active due to the strengthening of the westerly winds caused by the Southern Annular Mode, which has been increasing in the past two decades^16^. This phenomenon has strengthened the vertical exchanges of water masses in the SO, which supports the inference that there were remarkable increases in N_ex_ during the past 20 years in the Antarctic Intermediate Water and the Antarctic Bottom Water (Fig. S7).

#### Spatiotemporal distributions of ΔN_p_ and ΔN_ex_ over the Southern Ocean

Here, we used the same method as the previous sub-section to understand the distributions of ΔN_p_ (variation in internal N) and ΔN_ex_ over the entire SO. Considering the lack of observational data in the SO and the necessity for repeated observational data for the same location, we selected the observational data corresponding to the period 1990–1999 to represent the 1990s, 2000–2009 to represent the 2000s, and 2010–2017 to represent the 2010s. The data of each period were interpolated onto a common grid (see Supplementary Text S3). We used a grid with horizontal resolution of 1° × 1°, and 43 vertical layers with 50-m thickness from the surface to 500 m, 100-m thickness from 600 m to 1,500 m, and 200-m thickness from 1,700 m to the sea floor.

Seasonal differences between different cruises may affect our estimation. Owing to the severe environment of the SO, most of our observed data were collected in the warm period. In order to verify whether there was a significant difference between the data for cold period (for convenience, we call it wintertime) and warm period (for convenience, we call it summertime), we used the data of wintertime (April to October) and summertime (January to March) and calculated the average N_obs_ and N_p_ at each depth for these two durations (Fig. S8). We found that above the depth of 500 m the differences of both N_obs_ and N_p_ between the two seasons were ~3 μmol kg^−1^ as maximum; the corresponding differences at the depth of around 200 m became ~0.80 µmol kg^−1^, which was equal to the RMSE of our parameterization. These two periods did not show an obvious difference below the depth of 500 m. Thus, we concluded that the seasonal difference in the observational data does not significantly affect the spatiotemporal distributions of ΔN_ex_ along with the total water column inventory_._

**Figure 2:**
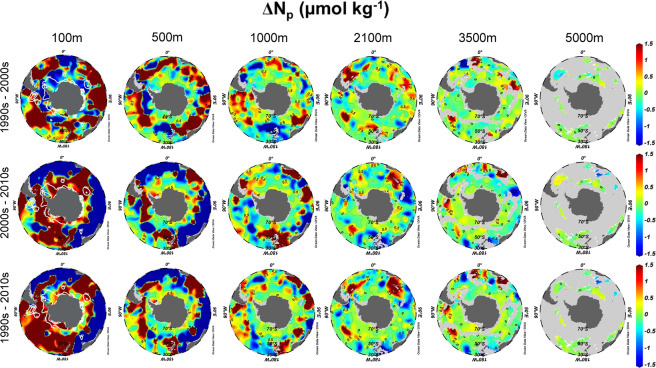
Horizontal distributions of the decadal change in N_p_ in the SO. Shown were ΔN_p_ from the 1990s to the 2000s (top row), 2000s to 2010s (middle row), and 1990s to 2010s (bottom row) at different depths, in units of µmol kg^–1^. Grey areas show the sea floors. White contour lines indicate the regions where the mixed layer is deeper than 100 m. This figure was drawn using Ocean Data View^31^.

**Figure 3:**
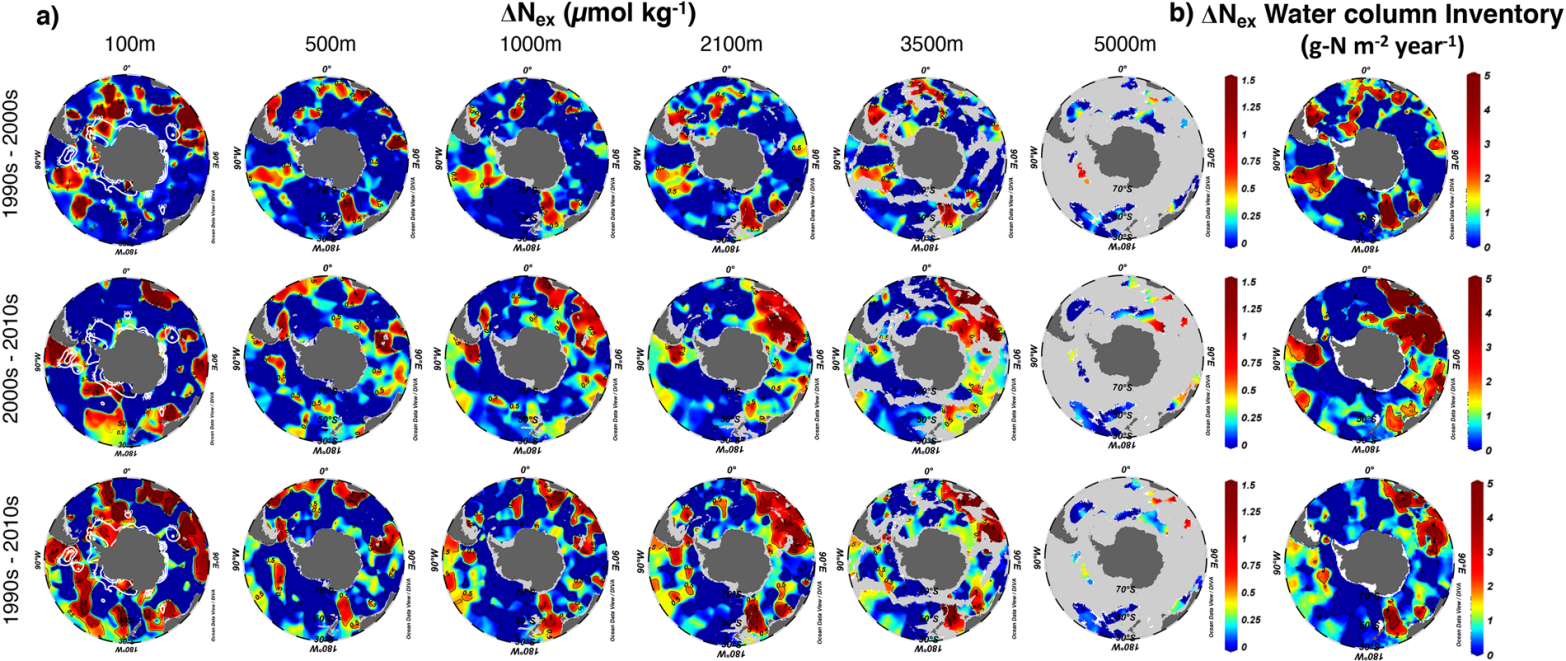
Horizontal distributions of the decadal change in N_ex_ in the SO. Shown along with the annual rate of change in the SO south of 30°S. (**a**) The decadal change in N_ex_ during the period from the 1990s to the 2000s (top row), 2000s to 2010s (middle row), and 1990s to 2010s (bottom row) at different depths. Scale was fixed from 0 to 1.5 µmol kg^–1^ to emphasize the increase in N_ex_. Grey areas show the sea floors. (**b**) Total water column inventories of ΔN_ex_ as the annual rate of change in N_ex_ over the same periods as in (**a**), in units of g-N m^−2^ year^−1^. Inventories were determined by integrating ΔN_ex_ from the surface to the depth of 5,900 m. White contour lines indicate the regions where the mixed layer is deeper than 100 m. This figure was drawn using Ocean Data View^31^.

The spatiotemporal distributions of ΔN_p_ and ΔN_ex_ are shown in Figs. 2 and 3(a), respectively. The spatiotemporal distribution of ΔN_p_ (Fig. 2) showed a large variation in the upper 1,000 m water column and it revolved around the Antarctic continent along with the Antarctic Circumpolar Current in the different time periods. This phenomenon may be due to the continuing enhanced nutrient-rich Circumpolar Deep Water upwelling derived from the strengthening of the Southern Hemisphere westerlies in recent decades^17,18^. Furthermore, ΔN_p_ became zero gradually with the increase of depth, implying that there is almost no nature-derived variation of N in the deeper water column. The distribution of ΔN_p_ showed no obvious difference between the Pacific, the Indian and the Atlantic sector of the SO.

In Fig. 3(a), the spatial distribution of ΔN_ex_ in the surface layer shows a tendency to diffuse along the continental coastal area to the open ocean (e.g. west coast of South America, southwest coast of South Africa, and south of Tasmania, Australia). The data for the continental shelf were removed to eliminate the uncertainty of river input in our parameterization construction based on the assumption that the riverine N_ex_ has little effect on the open ocean^1^ (see Supplementary Text S1). Jickells *et al*. (2017) found that approximately 75% of riverine N escapes beyond the shelf break and enters the open ocean, which may partly explain the significant rise in N_ex_ in the coastal region in our study^5^.

In terms of the temporal distribution of ΔN_ex_, the Indian sector has shown a remarkable growth in N_ex_ from the surface to the abyss during the period from the 2000s to the 2010s. By analyzing the spatiotemporal distribution, the reason for this can be attributed to the increase in anthropogenic nitrogen emission in developing countries such as India, China, and Southern Africa in the past decade^5,19,20^. According to the evaluation of the global meridional overturning circulation, the upwelling water in the surface North Pacific Ocean passes through the Strait of Malacca and reaches the northern Indian Ocean. Then, it goes south and flows into the Southern Ocean^7^. Meanwhile, the surface anthropogenic N is loaded on these waters along the coastal regions and brought to the Southern Ocean. Additionally, the enhancement of the Southern Annular Mode mentioned in the previous section can explain the increase in N_ex_ in the ocean interior.

We also estimated the total water column inventory of ΔN_ex_ from the surface to the sea floor in the SO (Fig. 3(b) and Table 1). During the 1990s to the 2010s, N_ex_ in the Pacific, Indian, and Atlantic sectors grew at the rate of 24 ± 1, 42 ± 1, and 0.02 ± 0 Tg-N year^−1^, respectively, and that for the entire SO grew at the rate of 67 ± 1 Tg-N year^−1^. Uncertainties were given by the standard error of gridding estimation (Table S6). The ΔN_ex_ in the Indian sector accounted for 63% of the increase in N_ex_ in the SO. We also found that the Atlantic Sector, which has the most active vertical circulation in the world, did not show a high ΔN_ex_. This may be because of the following two reasons: (1) the deviation caused by the seasonal differences in the surface data (Fig. S8); (2) the inflow of the deposition of N_ex_ from the Atlantic sector into the Indian sector due to the Antarctic Circumpolar Current, which also explains why ΔN_ex_ in the Indian sector is extremely high. In the Pacific Ocean, we mainly observed the accumulation of ΔN_ex_ in the surface layer (Fig. 3) due to the upwelling area with relatively old water age in the deep Pacific^21^. These results can be considered reasonable compared with the previous model predictions^1,5^.

**Table 1:** Total water column inventory of ΔN_ex_ in the SO.

Period	Pacific Sector	Indian Sector	Atlantic Sector	Southern Ocean
1990s–2000s	25 ± 1	−4 ± 1	17 ± 1	38 ± 2
2000s–2010s	24 ± 1	100 ± 2	−22 ± 1	102 ± 3
1990s–2010s	24 ± 1	42 ± 1	0.02 ± 0	67 ± 1

From the surface to 5,900-m depth south of 30°S during the 1990s – 2010s (Tg-N year^−1^) (Pacific Sector: 150°E – 60°W; Indian Sector: 20°E – 150°E; Atlantic Sector: 60°W – 20°E). The uncertainty is the value of the standard error divided by the average of each sector (see Table S6 for detail).

In an early study^1^, the deposition rate of N_ex_ to the global ocean was predicted as 67 ± 30 Tg-N year^−1^ in the 2000s, the upper limit of which was 96 Tg-N year^−1^ considering the potential impact of riverine input. By comparing the deposition rate with our data, we found that the SO had received 69% of the global oceanic N_ex_ input despite the SO covering only 29% of the global ocean surface area, which emphasizes the important role of the SO in integrating anthropogenic impacts in the global ocean.

## Conclusions

We presented the spatiotemporal distributions of ΔN_p_ and ΔN_ex_ in the SO from the 1990s to the 2010s using the simple parameterization of the predicted N along with the observed N (R^2^ = 0.97; RMSE = 0.80 µmol kg^−1^). In the Indian sector, which borders several developing countries, N_ex_ has grown at a rate of 42 ± 1Tg-N year^−1^, accounting for approximately 63% of the overall rate of increase of the SO (67 ± 1 Tg-N year^−1^). y comparing our result with the global deposition rate reported by Duce *et al*.^1^, the SO was found to receive approximately 70% of the global oceanic input of N_ex_ despite it covering only one-third of the global ocean area. In the future, a more detailed evaluation of N in the SO can be obtained by relying largely on ship-based observations and/or applying this parameterization method to autonomous biogeochemical Argo floats and CTD sensors^22–30^.

## Supplementary information

Supplementary information.

Supplementary information 2.
