# Supplementary material for: The Southern Ocean with the largest uptake of anthropogenic nitrogen into the ocean interior
Source: Sci Rep. 2020 Jun 1;10:8838. doi: 10.1038/s41598-020-65661-2 (PMC7264145; doi:10.1038/s41598-020-65661-2)

Supplementary Materials

Title

The Southern Ocean with the largest uptake of anthropogenic nitrogen into the ocean interior

**Authors**

Xianliang L. Pan^1^*, Bofeng F. Li^2^, Yutaka W. Watanabe ^2^

**Affiliations**

^1^ Graduate School of Environmental Science, Hokkaido University, Sapporo, Japan

^2^ Faculty of Environmental Earth Science, Hokkaido University, Sapporo, Japan

* Corresponding author: panxianliang@ees.hokudai.ac.jp

**Fig. S1**


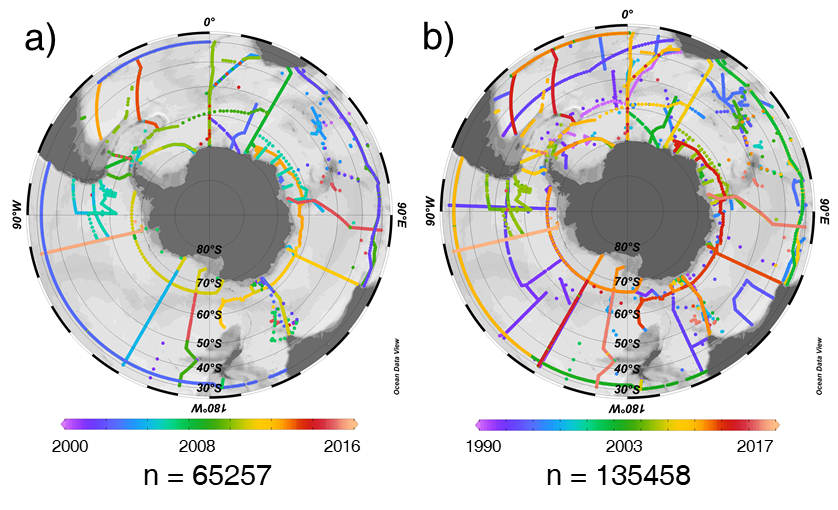


**
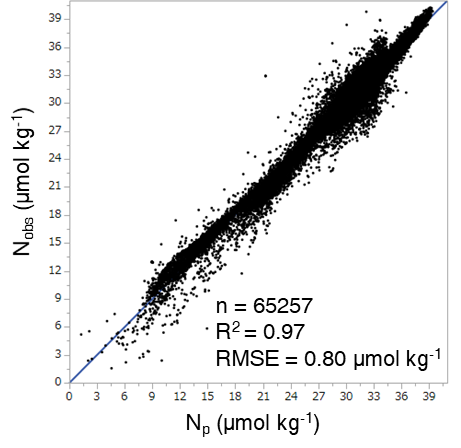
Fig. S2**


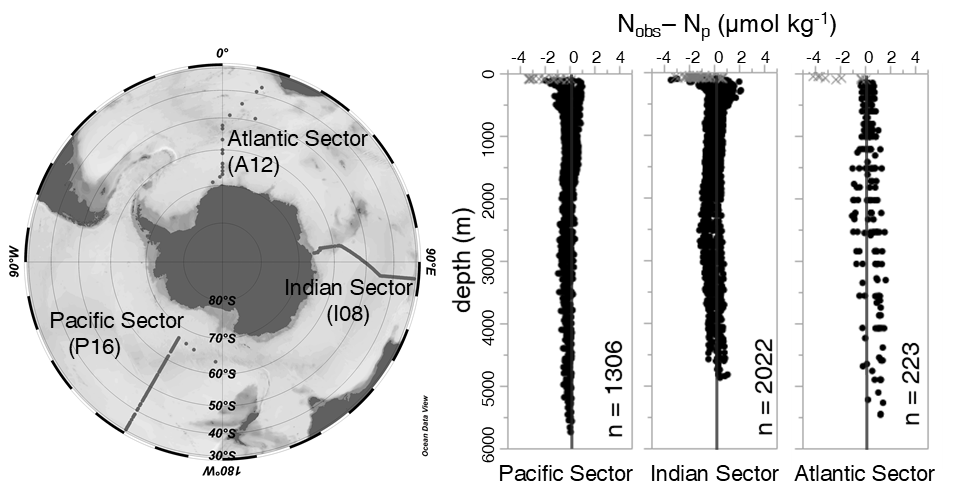
**Fig. S3**

**
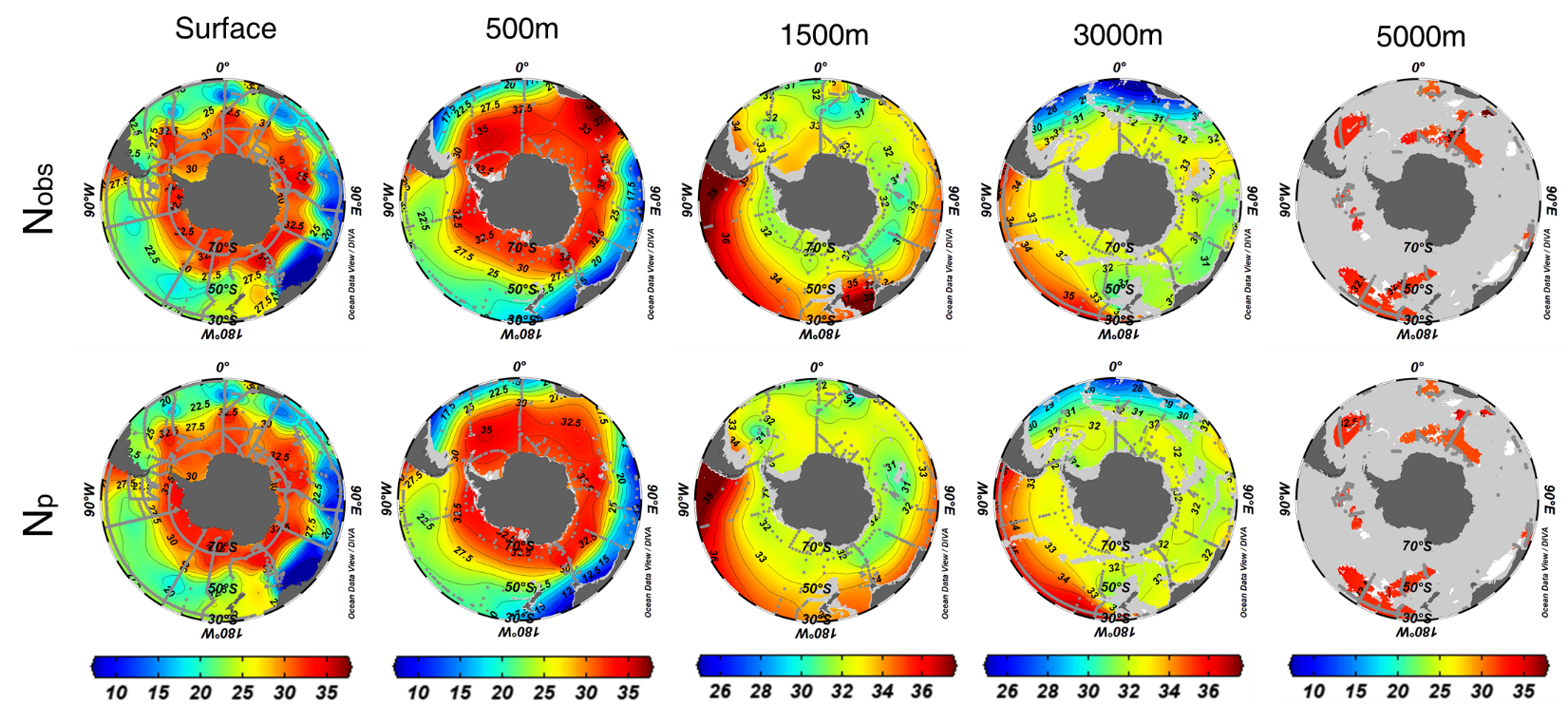
Fig. S4**

**Fig. S5**


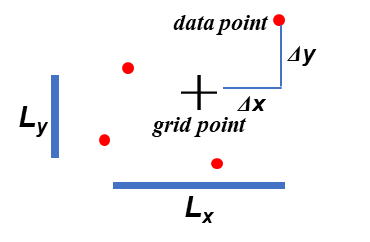


**Fig. S6**


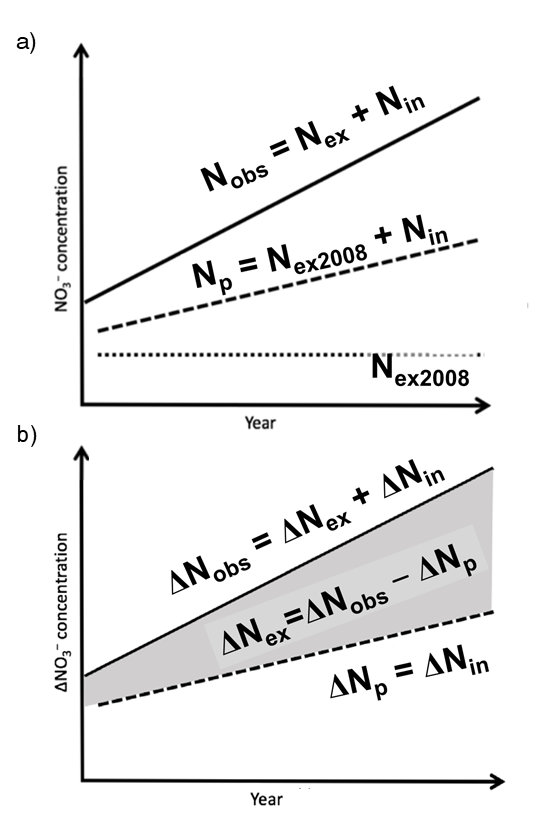


**
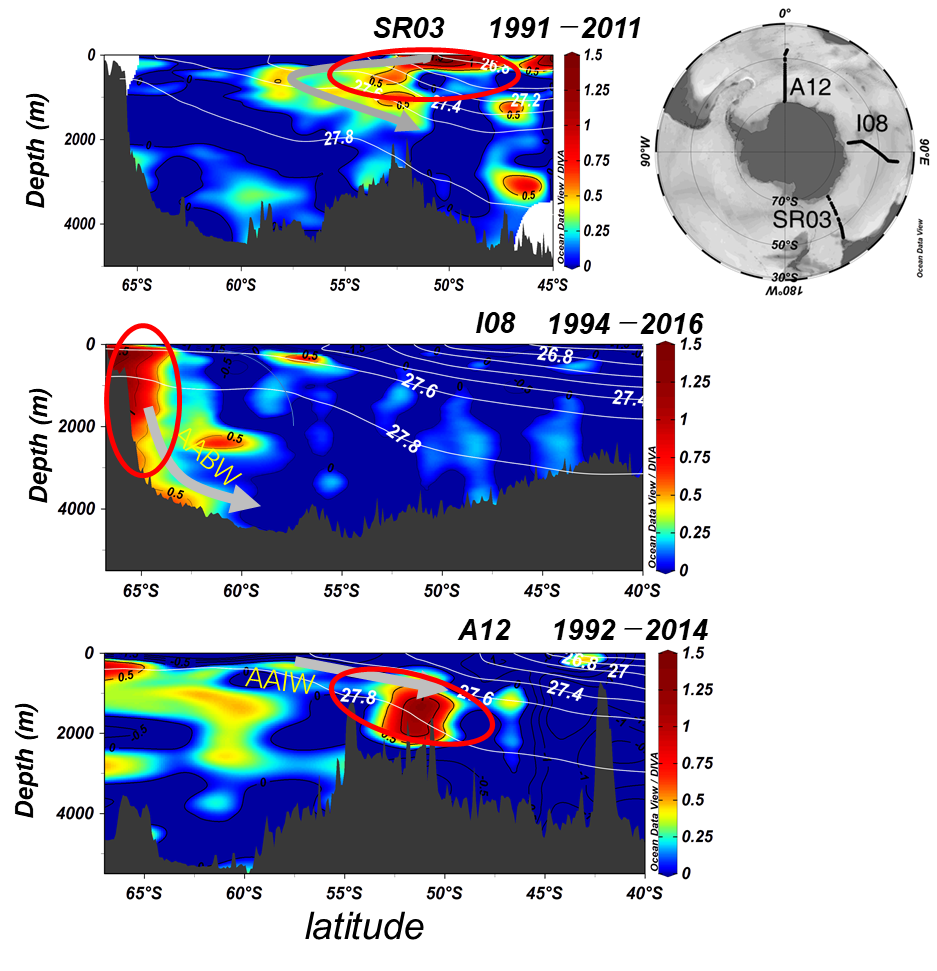
Fig. S7**

**Fig. S8**


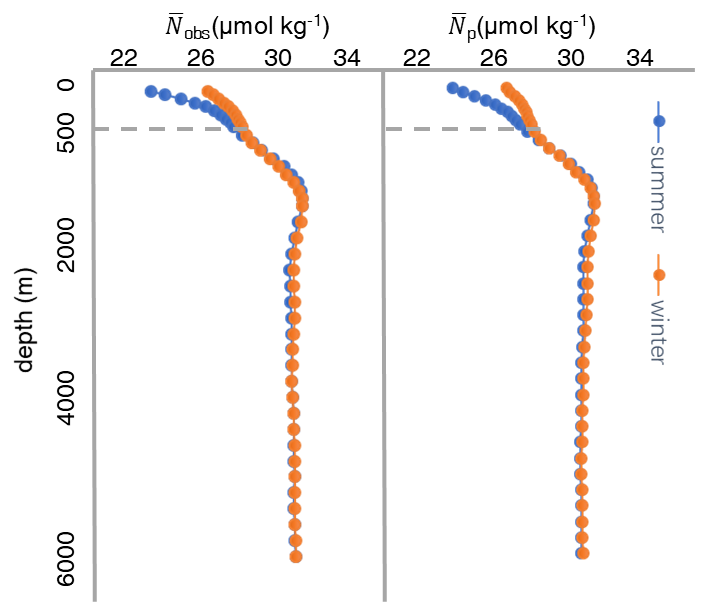

Supplement: Supplementary file 1 — Supplementary information. [file 41598_2020_65661_MOESM1_ESM.docx]
