# Supplementary material for: The Southern Ocean with the largest uptake of anthropogenic nitrogen into the ocean interior
Source: Sci Rep. 2020 Jun 1;10:8838. doi: 10.1038/s41598-020-65661-2 (PMC7264145; doi:10.1038/s41598-020-65661-2)
Supplement: Supplementary file 2 — Supplementary information 2. [file 41598_2020_65661_MOESM2_ESM.docx]

Supplementary Materials

Title

The Southern Ocean with the largest uptake of anthropogenic nitrogen into the ocean interior

**Authors**

Xianliang L. Pan^1^*, Bofeng F. Li^2^, Yutaka W. Watanabe ^2^

**Affiliations**

^1^ Graduate School of Environmental Science, Hokkaido University, Sapporo, Japan

^2^ Faculty of Environmental Earth Science, Hokkaido University, Sapporo, Japan

* Corresponding author: panxianliang@ees.hokudai.ac.jp

**Supplementary Text S1.**

**Data: Observational hydrographic data used in this study**

All the hydrographic observational data for nitrate (N), dissolved oxygen (DO), temperature (T), salinity (S), and pressure (Pr) were sourced from GLODAP v2 and CCHDO (https://cchdo.ucsd.edu/) ^12,13^. Information of the cruises in which we utilized the data is shown in Supplementary Tables S1 and S5. The EXPOCODE in these tables, which is constructed using the country code, platform code, and departure date, was used to identify each cruise ^22^. See the International Council for the Exploration of the Sea (ICES) library for deciphering the country and platform codes (http://www.ices.dk/marine-data/vocabularies/Pages/default.aspx). The quality flags of the World Ocean Circulation Experiment (WOCE) were used to check the quality of these data. In this study, we only used data with a quality flag of 2 (i.e. data value is acceptable). We used 65,257 data from 69 cruises from 2000 to 2016 for constructing the parameterization of N_p_, and 135,458 data from 101 cruises from 1990 to 2017 for estimating ΔN_ex_ (Fig. S1).

**Supplementary Text S2.**

**Method: Constraints and examination of parameterization of N_p_**

Data constraints for enhancing the accuracy of our parameterization are shown in Table S2. Here, we defined the Southern Ocean (SO) as the region south of 30°S. Our study mainly focused on atmospheric nitrogen deposition in the open ocean because the fluvial input process is very complicated ^5^. In addition, we defined the regions with bottom depths of less than 1500 m as the continental shelf and did not use these data in our parameterization. Considering that the lower limit of the current detection accuracy of nitrate is ~ 0.2 μmol kg^-1^, we only used N_obs_ data higher than this value. Although we used DO as a parameter to reflect the biological processes, DO in the surface mixed layer is directly affected by rapid air-sea exchange, which will have a significant impact on the regression; therefore, the observational data corresponding to the surface mixed layer were also removed. We defined the surface mixed layer as the water depth within a temperature difference of 0.5 ºC from the surface temperature (ΔT ≤ 0.5ºC) ^23^. In our study, the surface mixed layer had an average depth of ~60 m in the region of 30˚S south during 2000 to 2016, along with a few values deeper than 100 m encircled the Antarctic coastal region. The complex water mass composition of the SO posed great challenges to the parameterization construction. Through a series of comparative analyses, we found that the North Atlantic Deep Water (NADW) in the Northern Hemisphere and the Subantarctic Surface Water (SASW) north of 45°S showed significantly different characteristics of T and S when compared with the water masses formed in the Antarctic ^24^. To improve the accuracy of the regression, these two water masses were also not considered for constructing the parameterization.

The *F*-test is generally employed to determine the usefulness of each parameter in a multiple linear regression. A parameter is considered significant if its *F* value is greater than 2.4 ^25^. By constructing the predicted concentration of N (N_p_) in the SO (Eq. (1) and Fig. S2), T and S have *F* values of 1,426,953 and 213,532, respectively (Table S3). The increase in T and S results in decrease in N_p_ in Eq. (1), which is consistent with the stoichiometric relationship among N, T and S in seawater ^26,27^, indicating that T and S are significant for our regression. DO (O_2_), which has an *F* value of 903,917, also shows a negative correlation with N, reflecting the formation and consumption of N by primary production and respiration ^28^. At depths below 400 m, the N/–O_2_ ratios of remineralization appear to be constant at 16 ± 1/170 ± 10 (= 16/170 = 0.09) without considering denitrification (this ratio decreases to approximately 12 ± 2/170 ± 10 (= 12/170 = 0.07) when denitrification is considered) ^29^. In this study, the N/–O_2_ ratio was 0.09 (the regression coefficient for DO in Eq. (1)), indicating that denitrification was not included in our prediction. Standardized regression coefficients (β) were given to compare the contribution to N_p_ of each parameter. The closer the absolute value is to 1, the greater the contribution of the parameter is. Therefore, N_p_ was mainly controlled by T and DO in this study. We also investigated the presence of multicollinearity between variables by the variance inflation factor (VIF) (Fig. S3) and found that there was no multicollinearity in our parameterization. Generally, the variable is independent when the VIF is lower than 10 ^25^.

We used independent datasets to confirm the accuracy of our parameterization. We chose WOCE lines P16 (2014), I08 (2007), and A12 (2014), which covered the three main sectors of the SO. Differences between N_obs_ and N_p_ for each sector (Fig. S3 and Table S4) showed good agreements in the Pacific sector and the Indian sector. Deep water of the Atlantic Ocean (~2000 m) showed an obvious difference, which was probably caused by the movement of the anoxic water mass in the eastern tropical South Atlantic into the SO as North Atlantic Deep Water (NADW) ^30^. The mean absolute deviations (MAD) of the differences in the Pacific, Indian, and Atlantic sectors were 0.37, 0.47, and 0.64 µmol kg^-1^, respectively (Table S4). On comparing with the RMSE of our parameterization, these errors are acceptable in these three independent datasets.

**Supplementary Text S3.**

**Method: Gridding of data**

We used the weighted-average method provided by the Ocean Data View (ODV) software to interpolate our data onto a common grid (*c.f. Ocean Data View User’s Guide Version 5.2.1, 16.6.1,* https://odv.awi.de/documentation/) ^31^. To achieve this, we first constructed grids over the SO. The resolution of the grid can be assigned as per the requirement. In this study, the two-dimensional (2D) grid of cross sections was assigned a resolution of 0.5° $\times$10 m (latitude $\times$depth). The three-dimensional (3D) grid of the entire SO was constructed with a horizontal resolution of 1° $\times$1° (latitude $\times$longitude) and 43 layers with vertical resolution having 50-m thickness from the surface to 500 m, 100-m thickness from 600 m to 1500 m, and 200-m thickness from 1700 m to the sea floor.

After construction of the grid over the SO, the ODV allows us to use a simple weighted-averaging scheme to calculate the property estimation at every grid point (Eq. (S1)).

$c_{e}=\sum_{i} \alpha_{i}\cdot d_{i}/\sum_{i} \alpha_{i}$ (S1)

The weights of the data points $\alpha_{i}$ decrease exponentially with increasing distance between the data point and grid point: $\alpha_{i}=e^{-r}$, with $r={(\Delta x/L_{x})}^{2}+{(\Delta y/L_{y})}^{2}$, where $\Delta x$ and $\Delta y$ are the distances between the data point and grid point in the X and Y directions, respectively, and *L_x_* and *L_y_* are the separate averaging length-scales in the X and Y directions, respectively. The length scales of the X and Y directions in the 2D grid of the repeat-line estimation were both 50 permille. The length scale of the latitude and longitude in the 3D grid of the overall estimation was 50 permille, and the depth scale was 50 m.

**Supplementary Text S4.**

**Method: Estimation of external term**

Fig. S6 and Eqs. (S2–S5) explain the method to estimate $\Delta$N_ex_ based on the method proposed by Watanabe et al. (2018) ^10^. N_obs_ always contains both the internal term (N_in_) and the external term (N_ex_) (Eq. (S2)).

N_obs_ = N_ex_ + N_in_  (S2)

Thus, N_p_ predicted from our parameterization was already influenced by the external term due to use of modern observational data for constructing our parameterization. We defined N_ex_ contained in N_p_ as the average for the years 2000–2016 (N_ex2008_) because our parameterization is based on the observational data from 2000 to 2016 (Eq. (S3)).

N_p_ = N_ex2008_ + N_in_ (S3)

Arranging Eq. (S2) in the expression for N_ex_ and substituting Eq. (S3) into Eq. (S2), N_ex_ for an arbitrary year can be expressed by N_obs_, N_p_, and N_ex2008_ (Eq. (S4)).

N_ex_ = N_obs_ – N_in_

= N_obs_ – (N_p_ – N_ex2008_) (S4)

The only unknown term now is N_ex2008_, which is difficult to evaluate. Therefore, we made the key assumption that N_ex2008_ is constant with time, which makes it possible to cancel N_ex2008_ by calculating the time changes in the right and left sides of Eq. (S4) between two arbitrary different times (e.g. time ‘t1’ and time ‘t2’). We can estimate the change in N_ex_ ($\Delta$N_ex_) by the following equation;

ΔN_ex_ = N_ex(t2)_ – N_ex(t1)_

= N_obs(t2)_ – N_obs(t1)_ – [(N_p(t2)_–　N_p(t1)_) – (N_ex2008_– N_ex2008_)]

= ΔN_obs_ – ΔN_p_  (S5)

In summary, Eq. (S5) allows us to clarify ΔN_ex_ in the case where the observational data of N, DO, T, S, and Pr are repeatedly acquired for the same region. The appropriateness of this assumption has been proved in Supplementary Section 2 of the study by Watanabe et al. (2018) ^10^*.*

| **Table S1.** Information of cruises undertaken for constructing the parameterization of N_p_ from GLODAPv2 ^22^/CCHDO (https://cchdo.ucsd.edu/) during the period from 2000 to 2016 in this study. The map of cruise data is shown in Fig. S1(a). | | | |
| --- | --- | --- | --- |
| EXPOCODE | Sector | EXPOCODE | Sector |
| 09SS20090203 | Pacific | 33RR20160208 | Indian |
| 09FA20010524 | Pacific | 33RR20070204 | Indian |
| 096U20160426 | Pacific | 09AR20060102 | Indian |
| 49HH20011127 | Pacific | 09FA20000926 | Indian |
| 33RR20050109 | Pacific | 096U20160108 | Indian |
| 33RO20161119 | Pacific | 49NZ20130106 | Indian |
| 33RO20071215 | Pacific | 09AR20041223 | Indian |
| 316N20060130 | Pacific | 61TG20020206 | Indian |
| 316N20050821 | Pacific | 61TG20030217 | Indian |
| 740H20090203 | Pacific | 096U20160314 | Indian |
| 29HE20100208 | Pacific | 096U20150321 | Indian |
| 318M20091121 | Pacific | 09AR20080322 | Indian |
| 49NZ20030803 | Pacific | 09AR20070117 | Indian |
| 49NZ20121128 | Pacific | 09AR20120105 | Indian |
| 49NZ20071122 | Pacific | 09AR20071216 | Indian |
| 320620070205 | Pacific | 09AR20011029 | Indian |
| 320620110223 | Pacific | 35MF20030123 | Indian |
| 320620151208 | Pacific | 29HE20130320 | Atlantic |
| 09AR20110104 | Indian | 74JC20100319 | Atlantic |
| 74DI20041103 | Indian | 74JC20151217 | Atlantic |
| 33RR20080204 | Indian | 740H20090307 | Atlantic |
| 740H20081226 | Indian | 49NZ20031106 | Atlantic |
| 33RR20090320 | Indian | 06AQ20060825 | Atlantic |
| 74AB20020301 | Indian | 33RO20050111 | Atlantic |
| 74DI20041213 | Indian | 33RO20131223 | Atlantic |
| 35MF20020104 | Indian | 33RO20110926 | Atlantic |
| 35MF20040103 | Indian | 33RO20100308 | Atlantic |
| 35MF20010103 | Indian | 06AQ20101128 | Atlantic |
| 35MF20000117 | Indian | 06AQ20050122 | Atlantic |
| 35MF20000719 | Indian | 06AQ20021124 | Atlantic |
| 35MF20110114 | Indian | 06AQ20080210 | Atlantic |
| 35MF20050111 | Indian | 06AQ20141202 | Atlantic |
| 09AR20030103 | Indian | 35MF20080207 | Atlantic |
| 09AR20020126 | Indian | 90AV20041104 | Atlantic |
| 35MF20090103 | Indian |  |  |

| **Table S2.** Constraint conditions for parameterization of N_p_ in the SO. | | |
| --- | --- | --- |
| Constraints |  | Remarks |
| Bottom depth | > 1500 m | Removing continental shelf |
| Nitrate concentration | > 0.2 µmol kg^-1^ |  |
| Mixed layer depth | ΔT > 0.5 ℃ ^a^ | ΔT ≤ 0.5 ℃ was not used |
| Salinity | 34 – 35 |  |
| Water masses | Except NADW ^b^, SASW ^c^ | NADW: 34.8 < S < 35  1.5 ℃ < T < 4 ℃  SASW: T > 8 ℃ ^24^ |

^a^ Mixed layer depth is defined as the depth at which temperature (T) changes by a given threshold value (ΔT; here, ΔT = 0.5 ℃) relative to the temperature at the surface ^23^.

^b^ North Atlantic Deep Water

^c^ Subantarctic Surface Water; in this study, we only removed the northern part of SASW in the Pacific sector.

**Table S3.** Summary of the parameterization in our study for nitrate (N_p_) in the SO.

| Parameter | *F* ^a^ | *B* ^b^ | Standardized *β* ^c^ | VIF ^d^ |
| --- | --- | --- | --- | --- |
| Intercept | – | 394.3 | 0 | – |
| DO | 903,917 | –9.208$\times$10 ^-2^ | –0.70 | 1.23 |
| T | 1,426,953 | –1.534 | –0.80 | 1.26 |
| S | 213,532 | –9.862 | –0.37 | 1.63 |
| Pr | 4,830 | 2.029$\times$10^-4^ | 0.06 | 1.63 |

^a^ *F*-value with a significant level of α = 0.05; significant when *F*-value over 2.4 ^25^

^b^ Regression coefficient

^c^ Standardized regression coefficient

^d^ Variance Inflation Factor; indicates no multicollinearity when VIF is below 10 ^25^

**Table S4.** Mean Absolute Deviation (MAD) of difference between N_obs_ and N_p_ in three independent cruises along the Pacific, Indian, and Atlantic sectors in the SO during the period from 2000 to 2016.

| Region | Cruise | n ^a^ | MAD (µmol kg^-1^) |
| --- | --- | --- | --- |
| Pacific Sector | 320620140320 | 1,306 | 0.37 |
| Indian Sector | 33RR20070204 | 2,022 | 0.47 |
| Atlantic Sector | 06AQ20141202 | 223 | 0.64 |
| RMSE of N_p_ (µmol kg^-1^) | 0.80 | | |

^a^ Number of data points

| **Table S5.** Cruises used for estimating ΔN_ex_ from GLODAPv2 ^22^ /CCHDO. (https://cchdo.ucsd.edu/). The map of cruise data is shown in Fig. S1(b). | | | |
| --- | --- | --- | --- |
| EXPOCODE | Sector | EXPOCODE | Sector |
| 096U20160426 | Pacific | 316N19950310 | Indian |
| 06AQ20050122 | Pacific | 316N19950611 | Indian |
| 06AQ20080210 | Pacific | 33MW19950922 | Indian |
| 06MT19900123 | Pacific | 33RR20070204 | Indian |
| 09FA20010524 | Pacific | 33RR20080204 | Indian |
| 09SS20090203 | Pacific | 33RR20090320 | Indian |
| 29HE19951203 | Pacific | 33RR20160208 | Indian |
| 29HE19960117 | Pacific | 35MF19930123 | Indian |
| 316N19920502 | Pacific | 35MF19960220 | Indian |
| 316N19920901 | Pacific | 35MF19980121 | Indian |
| 316N19921006 | Pacific | 35MF19980818 | Indian |
| 316N19921204 | Pacific | 35MF19981205 | Indian |
| 316N19930222 | Pacific | 35MF19990104 | Indian |
| 316N20050821 | Pacific | 35MF20000117 | Indian |
| 316N20060130 | Pacific | 35MF20000719 | Indian |
| 318M20091121 | Pacific | 35MF20010103 | Indian |
| 31DS19940126 | Pacific | 35MF20020104 | Indian |
| 31DS19960105 | Pacific | 35MF20040103 | Indian |
| 31WT19910716 | Pacific | 35MF20090103 | Indian |
| 33RO20071215 | Pacific | 35UCKERFIXTS | Indian |
| 33RO20161119 | Pacific | 49HH19941213 | Indian |
| 33RR19971020 | Pacific | 49ZS19921203 | Indian |
| 33RR19971202 | Pacific | 61TG20020206 | Indian |
| 33RR20050109 | Pacific | 61TG20030217 | Indian |
| 49HG19950414 | Pacific | 74AB20020301 | Indian |
| 49HG19960412 | Pacific | 74DI19940219 | Indian |
| 49HH20011127 | Pacific | 74DI19950106 | Indian |
| 49NZ20030803 | Pacific | 74DI20041103 | Indian |
| 49NZ20071122 | Pacific | 74DI20041213 | Indian |
| 740H20090203 | Pacific | 06AQ19901117 | Atlantic |
| 90KD19920214 | Pacific | 06AQ19920521 | Atlantic |
| 32o619960906 | Pacific | 06AQ19920929 | Atlantic |
| 32o619970125 | Pacific | 06AQ19921203 | Atlantic |
| 32o620070205 | Pacific | 06AQ19960317 | Atlantic |
| 32o620110223 | Pacific | 06AQ19980328 | Atlantic |
| 32o620151208 | Pacific | 06AQ20021124 | Atlantic |
| 096U20150321 | Indian | 06AQ20060825 | Atlantic |

**Table S5.** Continued

| EXPOCODE | Sector | EXPOCODE | Sector |
| --- | --- | --- | --- |
| 096U20160108 | Indian | 06AQ20101128 | Atlantic |
| 096U20160314 | Indian | 06AQ20141202 | Atlantic |
| 35MF20110114 | Indian | 06MT19921227 | Atlantic |
| 49NZ20121128 | Indian | 29HE20100208 | Atlantic |
| 49NZ20130106 | Indian | 29HE20130320 | Atlantic |
| 09AR19910925 | Indian | 316N19940403 | Atlantic |
| 09AR19930404 | Indian | 32O619960503 | Atlantic |
| 09AR19941213 | Indian | 33RO20050111 | Atlantic |
| 09AR19960822 | Indian | 33RO20100308 | Atlantic |
| 09AR19980228 | Indian | 33RO20110926 | Atlantic |
| 09AR20011029 | Indian | 33RO20131223 | Atlantic |
| 09AR20030103 | Indian | 35A319950113 | Atlantic |
| 09AR20041223 | Indian | 35A319950221 | Atlantic |
| 09AR20060102 | Indian | 35MF20080207 | Atlantic |
| 09AR20071216 | Indian | 49NZ20031106 | Atlantic |
| 09AR20080322 | Indian | 740H20081226 | Atlantic |
| 09AR20110104 | Indian | 740H20090307 | Atlantic |
| 09AR20120105 | Indian | 74DI19921222 | Atlantic |
| 09FA19930624 | Indian | 74DI19930206 | Atlantic |
| 09FA19941112 | Indian | 74JC20100319 | Atlantic |
| 09FA19960507 | Indian | 74JC20151217 | Atlantic |
| 09FA20000926 | Indian | 32o619960503 | Atlantic |
| 316N19941201 | Indian | 323o19940104 | Atlantic |
| 316N19950124 | Indian |  |  |

**Table S6.** Uncertainty of the estimation of ΔN_ex_ water column inventory in the SO.

| Period |  | Pacific Sector | Indian Sector | Atlantic Sector | Southern Ocean |
| --- | --- | --- | --- | --- | --- |
|  | grid number | 5,309 | 4,365 | 2,802 | 12,476 |
| 1990s–2000s | average | 0.0044 | 0.0009 | 0.0055 | 0.3652 |
|  | SE | 0.00016 | 0.00021 | 0.00022 | 0.01628 |
|  | *uncertainty* | 4% | 22% | 4% | 4% |
| 2000s–2010s | average | 0.0042 | 0.0230 | 0.0069 | 0.9354 |
|  | SE | 0.00016 | 0.00036 | 0.00033 | 0.02455 |
|  | *uncertainty* | 4% | 2% | 5% | 3% |
| 1990s–2010s | average | 0.0043 | 0.0107 | 0.0061 | 0.6186 |
|  | SE | 0.00016 | 0.00028 | 0.00027 | 0.01189 |
|  | *uncertainty* | 4% | 3% | 4% | 2% |

^a^ Grid number in each sector when calculated the ΔN_ex_ water column inventory.

^b^ Average of the inventory value of each grid point in unit of Tg-N year^-1^.

^c^ Standard error of the inventory value of each grid point in unit of Tg-N year^-1^.

^d^ Uncertainty of the ΔN_ex_ water column inventory, which is calculated by SE/average in each sector.

**Fig. S1.** Map of cruise data used in this study. The color shows the date of each cruise. The notation ‘n’ indicates the number of data points. **(a)** Cruise data during the period from 2000 to 2016 used for constructing the parameterization in this study. **(b)** Cruise data used for estimating ΔN_ex_. This figure was drawn using Ocean Data View ^31^.

**Fig. S2.** Relationship between predicted nitrate (N_p_) and observed nitrate (N_obs_) from 0 – 6,000 m south of 30°S during the period from 2000 to 2016 in the SO. The notation ‘n’ indicates the number of data points; R^2^ indicates the coefficient of determination; RMSE indicates the root-mean-square error. The number of data used in the Pacific sector, Indian sector, and Atlantic sector are 29,391, 22,566, and 13,300, respectively. (Pacific Sector: 150° E–60°W; Indian Sector: 20°E–150°E; Atlantic Sector: 60°W–20°E).

**Fig. S3.** The differences between N_obs_ and N_p_ (N_obs_ – N_p_) for the data used in the three independent cruises; P16 (2014) for the Pacific sector; I08 (2007) for the Indian sector; A12 (2014) for the Atlantic sector. Grey crosses indicate data within 100 m from the surface.

**Fig. S4.** Comparison between the distributions of N_obs_ (upper) and N_p_ (below) in the SO of 30°S south at surface, 500 m, 1,500 m, 3,000 m and 5,000 m south of 30°S between 2000 and 2016. Color of the map shows the nitrate concentration in unit of µmol kg^-1^. Gray dots show the position of observed data used for the parameterization. This figure was drawn using Ocean Data View ^31^.

**Fig. S5.** Weighted averaging of data values at a grid point, redrawn based on ODV User’s Guide Version 5.2.0, Fig. 16-2. (https://odv.awi.de/documentation/) ^31^.

**Fig. S6.** Schematic illustrations of our method to estimate ΔN_ex_. In (**a**), the solid line indicates the observed nitrate value (N_obs_); the dashed line indicates the predicted nitrate value (N_p_); the short-dashed line indicates the N_ex_ in 2008 (N_ex2008_), which can be assumed to be a constant with time. In (**b**), the solid line indicates the change in N_obs_ (ΔN_obs_) between arbitrary different years; the dashed line indicates the change in N_p_ (ΔN_p_) between arbitrary different years; the shadow area shows the difference between ΔN_obs_ and ΔN_p_, which is the change in N_ex_ (ΔN_ex_) between arbitrary different years.

**Fig. S7.** Cross section distributions of ΔN_ex_ in SR03 (top), I08 (middle), and A16 (bottom) from the 1990s to the 2010s. White solid lines indicate the potential density of seawater. Grey arrows show the supposed trajectory of ΔN_ex_ inflow. Red circles emphasize the regions where N_ex_ increased remarkably. This figure was drawn using Ocean Data View ^31^.

**Fig. S8.** Vertical distributions of average N_obs_ and N_p_ for summertime (blue circle) and wintertime (orange circle) based on the data from the 1990s to the 2010s south of 30°S. Differences between summertime and wintertime indicate seasonal variations.
